# Role of oxytocin in the control of stress and food intake

**DOI:** 10.1111/jne.12700

**Published:** 2019-03-19

**Authors:** Tatsushi Onaka, Yuki Takayanagi

**Affiliations:** ^1^ Division of Brain and Neurophysiology Department of Physiology Jichi Medical University Shimotsuke‐shi Japan

**Keywords:** active stress coping, food intake, oxytocin, stress

## Abstract

Oxytocin neurones in the hypothalamus are activated by stressful stimuli and food intake. The oxytocin receptor is located in various brain regions, including the sensory information‐processing cerebral cortex; the cognitive information‐processing prefrontal cortex; reward‐related regions such as the ventral tegmental areas, nucleus accumbens and raphe nucleus; stress‐related areas such as the amygdala, hippocampus, ventrolateral part of the ventromedial hypothalamus and ventrolateral periaqueductal gray; homeostasis‐controlling hypothalamus; and the dorsal motor complex controlling intestinal functions. Oxytocin affects behavioural and neuroendocrine stress responses and terminates food intake by acting on the metabolic or nutritional homeostasis system, modulating emotional processing, reducing reward values of food intake, and facilitating sensory and cognitive processing via multiple brain regions. Oxytocin also plays a role in interactive actions between stress and food intake and contributes to adaptive active coping behaviours.

## 1 INTRODUCTION

Oxytocin, a nonapeptide, is mainly synthesised in magnocellular paraventricular and supraoptic neurones of the hypothalamus, in parvocellular paraventricular neurones and, in part, in neurones of the bed nucleus of the stria terminalis in mammals.^1^, ^2^ Hypothalamic parvocellular oxytocin‐synthesising neurones project their axons to various brain regions, including the spinal cord, dorsal vagal complex, nucleus tractus solitarius, rostral ventral medulla, ventral tegmental area and hypothalamus. Magnocellular oxytocin neurones, which mainly project their axon terminals to the neurohypophysis, also extend their axon collaterals to some brain regions, including the amygdala, lateral septum and nucleus of the horizontal limb of the diagonal band.^3^ Reproduction‐related stimuli, including mating, parturition and lactation, have been shown to activate magnocellular oxytocin neurones and facilitate oxytocin release from axon terminals of the neurohypophysis into the peripheral circulation. Oxytocin is released not only from neurohypophyseal axon terminals, but also from dendrites or somas of hypothalamic magnocellular oxytocin neurones within the hypothalamus^4^ or the medial amygdala.^5^ Oxytocin is also released from axon terminals in intracerebral target regions. Oxytocin has been shown to have multiple functions^6^, ^7^ by acting mainly on the oxytocin receptor and possibly on the vasopressin receptor.^8^

In this review, we focus on the role of oxytocin in the control of stress responses and food intake. In the last part, we briefly review several hypotheses for explaining multiple oxytocin functions to control social behaviour, as well as stress‐coping behaviours.

## 2 OXYTOCIN AND STRESS

### 2.1 Activation of oxytocin neurones after stressful stimuli

Stressful stimuli induce stereotypical alarm responses, especially in the neuroendocrine system, autonomic nervous system and immune system and in behaviours, and influence health conditions in humans.^9^ Some stressful stimuli, including noxious stimuli,^10^ conditioned fear stimuli,^11^ social defeat,^12^, ^13^, ^14^, ^15^, ^16^ immobilisation stress,^17^ shaker stress^18^ and forced swimming^19^, ^20^, as well as administration of lipopolysaccharide^21^ and interleukin‐1,^22^ activate oxytocin‐synthesising neurones in the hypothalamus and facilitate oxytocin release into the plasma or within the brain of mice or rats.^23^, ^24^ Exercise has also been shown to facilitate oxytocin release in horses.^25^ Oxytocin is released into peripheral circulation and within the brain. Parallel oxytocin release within the hypothalamus and into the peripheral circulation has been shown during forced swimming stress^20^ and shaker stress in rats.^18^ However, dissociations between peripheral and central release have also been reported in rats during social defeat stress,^15^ after adrenalectomy^20^ and after administration of α‐melanocyte‐stimulating hormone (α‐MSH).^26^ In humans, stressful stimuli such as physical running,^27^, ^28^, ^29^, ^30^ exposure to psychological stress induced by the Trier social stress test, which consists of public speaking and mental arithmetic in front of unknown jury members,^28^, ^31^ and interpersonal distress^32^ increase oxytocin in plasma or saliva, although no significant increase after psychological stress in urine of children^33^ or in plasma of female participants^34^ has also been reported.

Activation of oxytocin neurones in response to conditioned fear stimuli is mediated, at least in part, by A2 noradrenergic/prolactin‐releasing peptide (PrRP) neurones in the nucleus tractus solitarius of the medulla oblongata^35^ and by neurones of the medial amygdala,^36^ whereas activation of oxytocin neurones after noxious stimuli has been suggested to be mediated by A1 noradrenergic neurones in the medulla oblongata.^10^ The hypothalamic paraventricular and supraoptic nuclei have also been shown to receive inputs not only from noradrenergic neurones in the medulla oblongata, but also from neurones in other stress‐related areas, including the amygdala, septum, bed nucleus of the stria terminalis, dorsomedial hypothalamus, parabrachial nucleus and raphe nuclei.^37^ Further studies are needed to clarify the roles of these regions in activation of oxytocin neurones in response to stressful stimuli.

### 2.2 Roles of oxytocin in stress responses: attenuation or facilitation of stress responses

Oxytocin has been shown to modulate stress responses in the neuroendocrine system, autonomic nervous system and immune system and in behaviours. In laboratory animals and humans, oxytocin has been reported to reduce activity of the hypothalamic‐pituitary adrenal axis,^38^, ^39^, ^40^, ^41^, ^42^ attenuate inflammation^43^, ^44^ and reduce anxiety‐related behaviours.^7^, ^39^, ^45^, ^46^, ^47^, ^48^ Oxytocin‐deficient female mice have been shown to exhibit enhanced corticosterone release in response to shaker stress, enhanced novel environment‐induced hyperthermia and increased anxiety‐related behaviours in an elevated plus maze test.^49^, ^50^ These findings suggest that endogenous oxytocin has inhibitory actions on activity of the hypothalamic‐pituitary‐adrenal axis, sympathetic activity and anxiety‐related behaviour during exposure to stressful stimuli.^51^

Social relationships such as bonds between couples, mother‐offspring relationships and same‐sex platonic adult relationships have protective actions against adverse environments and evoke beneficial effects on health status.^52^, ^53^ This social interaction‐induced attenuation of stress responses is called social buffering.^54^ Social interactions ameliorate stress responses via sensation of visceral, tactile, olfactory, auditory and visual cues. Oxytocin has been suggested to be involved in social buffering in the neuroendocrine system and in behaviours of laboratory animals and humans.^55^

During sexual interactions, hypothalamic oxytocin neurones are activated in male rats.^56^ Oxytocin neurones are also activated in female rats under a paced mating condition in which females have the initiative to control sexual interactions.^57^ Experiments with oxytocin receptor antagonists have shown that endogenous oxytocin attenuates anxiety‐related behaviour in these mating conditions.

Oxytocin has also been suggested to reduce anxiety‐related behaviour or neuroendocrine stress responses during non‐mating social interactions in humans,^58^, ^59^ chimpanzees^60^ and prairie voles.^61^ In human children, comfort interactions with their mothers have been shown to increase urinary oxytocin and reduce an increase in cortisol after psychological stress.^33^ In chimpanzees, post‐conflict affiliations have been shown to increase urinary oxytocin.^60^ These findings suggest that activation of oxytocin neurones by social support reduces stress responses. In male prairie voles, it has been shown that oxytocin induces consolation behaviour towards their distressed female partners, resulting in induction of anxiolytic actions in both the giver and receiver of consolation.^62^ Oxytocin receptor‐dependent consolation behaviour toward distressed partners has also been reported in mandarin voles.^63^

Oxytocin has also been shown to have analgesic effects.^64^ Oxytocin neurones are activated after noxious stimuli^10^ and oxytocin acts on multiple sites, including the spinal cord and dorsal root ganglia, to induce analgesia.^65^, ^66^, ^67^, ^68^

As stated above, oxytocin has anti‐stress actions in many cases. However, oxytocin has also been shown to have anxiogenic effects in some conditions.^69^, ^70^ For example, it has been shown in humans that administration of oxytocin increases perceived social stress,^71^ facilitates fear conditioning,^72^ enhances startle reflex to unpredictable shocks^73^ and potentiates acoustic startle responses after exposure to negative emotional pictures.^74^

In a mouse model of social defeat stress, oxytocin has also been shown to facilitate stress‐related behaviours. Social defeat stress activated oxytocin‐synthesising neurones in the hypothalamus and bed nucleus of the stria terminalis, and oxytocin receptor‐deficient male mice showed a deficit in facilitation of social defeat posture observed during repeated social defeat, suggesting that the oxytocin receptor facilitates expression of social defeat posture.^13^ A local oxytocin receptor antagonist has also been shown to reduce social avoidance after social defeat stress in female mice, suggesting involvement of the oxytocin receptor in social avoidance.^12^

Oxytocin may also induce negative emotion via facilitation of negative emotional contagion. Emotional states, especially negative emotions, can be transmitted among in‐group familiar members. This is called emotional contagion. Intranasal oxytocin application and chemogenic activation of hypothalamic oxytocin neurones have been shown to enhance socially transmitted fear, whereas an oxytocin antagonist impairs observational fear in male mice, suggesting that oxytocin facilitates fear contagion.^75^

In summary, oxytocin has been shown to attenuate stress responses in the hypothalamo‐pituitary‐adrenal axis and the autonomic nervous system and in behaviour, especially in non‐competitive comfort situations. When facing stressful or threatening situations, seeking available social support by increasing social salience as a result of activation of the oxytocin system is an adaptive strategy when social support is expected. Indeed, oxytocin antagonist administration to couples of marmosets has been shown to decrease the time spent together in a novel‐housing stress condition.^42^ It is possible that oxytocin attenuates stress responses, at least in part, by inducing social support‐seeking behaviour and, as a result, reducing the risk of stressful stimuli.^76^ On the other hand, oxytocin appears to augment stress responses in socially aversive situations. When environments are severe with no hope of social support, augmentation of stress responses in the autonomic and behavioural systems may be more adaptive for coping with the situations.

### 2.3 Sites of action of oxytocin with respect to modulating stress responses

Oxytocin acts mainly on the oxytocin receptor. The oxytocin receptor is located in multiple brain areas that modulate stress responses, including the prefrontal cortex, limbic area, hypothalamus and medulla oblongata. Detailed downstream mechanisms of oxytocin remain to be clarified. However, stressful stimuli have been shown to activate oxytocin receptor‐expressing neurones in several distinct brains regions. For example, social defeat stress activated oxytocin receptor‐expressing neurones in the insula cortex, amygdala, paraventricular thalamic nucleus, posterior intralaminar thalamic nucleus, ventrolateral part of the ventromedial hypothalamus and ventrolateral periaqueductal gray,^13^ which have been shown to modulate autonomic or behavioural stress responses.

#### 2.3.1 Medial prefrontal cortex

The medial prefrontal cortex of rodents consists of three parts, consisting of the anterior cingulate, prelimbic prefrontal cortex and infralimbic prefrontal cortex, and coordinates integrative responses in autonomic and behavioural systems to adapt to stressful environments.^77^

Activation of the oxytocin receptor in the medial prefrontal cortex has been shown to have anxiolytic actions (Table 1). Bilateral oxytocin administration into the prelimbic medial prefrontal cortex of rats has been reported to reduce anxiety‐related behaviour in an elevated plus maze test and an open field test and facilitate social interaction behaviours toward unfamiliar conspecifics.^78^ Oxytocin administration into the infralimbic region of the medial prefrontal cortex has also been shown to facilitate extinction of contextual conditioned fear and, as a result, induce attenuated freezing behaviour in response to conditioned fear stimuli in rats.^79^ Oxytocin has been shown to activate oxytocin receptor‐expressing GABAergic/corticotrophin‐releasing factor binding protein (CRFBP) interneurones in the medial prefrontal cortex, to induce anxiolytic actions in male mice via release of CRFBP, which suppresses activation of CRF receptor 1 (CRFR1)‐expressing neurones in layer 2/3,^80^ and to induce social approach toward male mice in female mice.^81^ Oxytocin in the infralimbic prefrontal cortex has also been shown to mediate social interaction‐induced facilitation of extinction of conditioned fear in rats.^82^

**Table 1:** Roles of the oxytocin receptor in limbic areas in the control of anxiety‐related behaviours or conditioned fear responses

	Anxiety	Acquisition (treatments before fear conditioning)	Recall (treatments before recall test)	Extinction	
				Acquisition (treatments before recall test)	Consolidation (treatments after recall test
Medial prefrontal cortex					
Prelimbic	↓^1^ male rats, EPM, OT^78^				
	↓^1^ male mice, EPM OF, optogenetic activation of OTR neurones, conditional OTR deletion^80^				
Infralimbic	±^2^ male rats, EPM, OT^78^		↓^1^ male rats, contextual CF, OTa^82^	↓^1^ male rats, contextual CF, OTa^82^	↓^1^ male rats, contextual CF, OT OTa^79^
					±^2^juvenile male rats, contextual CF, OTa^105^
Basolateral amygdala		↓^1^ male rats, contextual CF, OT^100^	↓^1^ male rats, contextual CF, OT^100^	↓^1^ male rats, contextual CF, OT^100^	±^2^ male rats, contextual CF, OT^100^
		↑^1^ male rats, contextual CF, OT OTa^79^			↑^3^ juvenile male rats, contextual CF, OTa^105^
		±^2^ juvenile male rats, contextual CF, OTa^105^			↓^1^ male rats, contextual CF, OTa^79^
					±^2^ male rats, contextual CF, OTa^105^
Central amygdala	↓^1^ male mice, EZM, OTA^102^	↓^1^ male rats, contextual CF, OT OTa^79^, ^100^, ^105^	↓^1^ female rats, contextual CF, optogenetic activation of OT afferents^3^	↑^3^ male rats, contextual CF, OT^100^	↑^3^ male rats, contextual CF, OT^100^
	↓^1^ female mandarin voles, EPM OF, OT OTA^285^	↓^1^ male rats, contextual CF, OT^100^	↓^1^ male rats, contextual CF, OTa^104^		±^2^ male rats, contextual CF, OT OTa^79^
		↓^1^ male rats, contextual CF, OTa^105^	↑^3^ male rats, contextual CF, OT OTa OTA^100^		±^2^ juvenile male rats, contextual CF, OTa^105^
		↑^3^ juvenile male rats, Contextual CF, OTa^105^			
Bed nucleus of stria terminalis	↑^4^ female California mice, social defeat‐induced social avoidance, OTA^12^	↑^3^ male rats, cued fear‐potentiated acoustic startle, OTA^111^			
Lateral septum	±^2^ male mice, EPM, OT^95^	±^2^ lactating mice, social fear conditioning, OTA^96^	↓^5^ male mice, social fear conditioning, OT^95^	↓^5^ male mice, social fear conditioning, OT^95^	
			↓^5^ lactating or virgin mice, social fear conditioning, OT OTA OTR overexpression chemogenetic silencing of OT afferents^96^		
			±^2^ female mice, cued CF, OTR overexpression^96^		

±^2^, no significant change in anxiety‐related behaviours, freezing behaviour, or social investigation; ↑^3^, increased anxiety‐related behaviours or freezing behaviour (oxytocin having anxiogenic actions); ↑^4^, facilitation of social defeat‐induced social withdrawal (oxytocin inducing social avoidance); ↓^1^, reduced anxiety‐related behaviours or freezing behaviour (oxytocin having anxiolytic actions); ↓^5^, blockade of conditioned fear‐induced suppression of social investigation behaviour (oxytocin having anxiolytic actions); CF, conditioned fear; EPM, elevated plus maze test; EZM, elevated zero maze test; OF, open field test; OT, oxytocin; OTa, oxytocin receptor agonist; OTA, oxytocin receptor antagonist; OTR, oxytocin receptor.

In the experiments with conditioned fear, microinjections of oxytocin, oxytocin receptor agonists or oxytocin receptor antagonists or optogenetic manipulations were performed before fear conditioning training (pairings of conditioned stimuli and electric foot shocks), after fear conditioning training, before recall of conditioned responses (application of conditioned stimuli), and after recall of conditioned responses.

Reported functions of activation of the oxytocin receptor, sex and species, tests and local manipulations of oxytocin systems (optogenetic activation or microinjections of oxytocin, oxytocin receptor agonists or oxytocin receptor antagonists) are shown. To examine the roles of oxytocin in acquisition of fear conditioning, oxytocin manipulations were performed before fear conditioning and responses to conditioned stimuli were investigated. To examine the roles of oxytocin in recall of fear conditioning, oxytocin manipulations were performed before the recall test (application of conditioned fear). To examine the roles of oxytocin in acquisition of extinction learning, oxytocin manipulations were performed before the recall test. To examine the roles of oxytocin in consolidation of extinction learning, oxytocin manipulations were performed after the recall test.

In humans, intranasal oxytocin administration has been shown to increase activity of the prefrontal cortex and functional connectivity of the prefrontal cortex with the posterior cingulate cortex and precuneus, to decrease amygdala activity, and to facilitate extinction of fear conditioning.^83^

Oxytocin may also induce anxiolytic actions by evoking consolation behaviour via the oxytocin receptor in the anterior cingulate. Consolation behaviour of male prairie voles and mandarin voles toward distressed female partners has been shown to be mediated by the oxytocin receptor in the anterior cingulate.^62^, ^63^ Consolation reduces anxiety‐related behaviour and activity of the hypothalamo‐pituitary adrenal axis in both givers and receivers of consolation.

#### 2.3.2 Nucleus accumbens

Male prairie voles show depression‐like behaviour (floating in a forced swimming test and tail suspension test) after separation from their female partners. Reduction of oxytocin transmission in the nucleus accumbens has been shown to be involved in this depression‐like behaviour in prairie voles.^84^ The oxytocin receptor in the nucleus accumbens has been shown to be up‐regulated via an epigenetic regulation, involving an increase of histone acetylation at a promoter region of the oxytocin receptor gene, after mating. This up‐regulation has been shown to facilitate partner preference formation in female^85^ and male^86^ prairie voles. Separation from partners reduces oxytocin synthesis in the hypothalamus and expression of the oxytocin receptor in the nucleus accumbens. Activation of the CRF receptor 2 in the nucleus accumbens suppresses local oxytocin release. Oxytocin administration into the nucleus accumbens shell has been shown to reverse separation‐induced passive coping behaviour, and local knockdown of the oxytocin receptor induces depression‐like passive coping behaviour, suggesting that separation increases CRFR2 signalling, which lowers oxytocin transmission within the nucleus accumbens, resulting in depression‐like behaviour. Consistent with the view of attenuating action of the oxytocin receptor on behavioural stress responses, oxytocin receptor binding in the nucleus accumbens in prairie voles has been shown to be reduced by immobilisation stress, and the presence of their partners dampens both reduction of the oxytocin receptor and anxiety‐related behaviour.^87^

#### 2.3.3 Hypothalamic paraventricular nucleus

Bilateral infusions of oxytocin into the hypothalamic paraventricular nucleus of adult male rats^88^ and of female prairie voles^89^ have been shown to reduce anxiety‐related behaviour in an elevated plus maze test. The paraventricular oxytocin receptor is also involved in social buffering actions. Microinjection of oxytocin into the hypothalamic paraventricular nucleus has been reported to reduce stress responses of anxiety‐related behaviours and the hypothalamic‐adrenal axis, whereas that of an oxytocin antagonist impairs the social buffering actions on behavioural and neuroendocrine responses in female prairie voles.^61^

Activation of GABAergic neurones has been shown to be involved in the inhibitory actions of oxytocin on anxiety‐related behaviour and the hypothalamic‐pituitary‐adrenal axis. Pharmacological blockade of the GABA_A_ receptor has been reported to block the actions of oxytocin in prairie voles.^89^ On the other hand, it has been reported that there is no mRNA expression of the oxytocin receptor in parvocellular hypothalamic CRH‐positive neurones of rats^90^, ^91^ and in hypothalamic CRH neurones of mice,^92^ and oxytocin has been shown to reduce the frequency of the spontaneous excitatory postsynaptic current in some CRH neurones of mice.^93^ These findings suggest that oxytocin suppresses the activity of hypothalamic CRH neurones also by suppressing excitatory synaptic transmission onto CRH neurones presynaptically.

#### 2.3.4 Lateral septum

Deficiency of the oxytocin receptor in the lateral septum has been shown to reduce both social defeat‐induced facilitation of freezing behaviour in response to contextual fear and social buffering‐induced reduction of behavioural fear responses, suggesting that activation of the septal oxytocin receptor enhances social memory of negative or positive social interactions and, as a result, facilitates or reduces behavioural fear responses in mice.^94^

On the other hand, the oxytocin receptor in the lateral septum has been shown in mice to have behaviourally anxiolytic actions in certain conditions that cannot be explained by the facilitative action of oxytocin on social memory. Oxytocin injected into the dorsolateral septum has been shown to reduce social avoidance behaviour after social fear conditioning in which electric foot‐shocks were applied during investigation of a conspecific with social contact.^95^ Interestingly, lactating mice, whose oxytocin systems are activated, do not show this social avoidance. This avoidance has been shown to be recovered by local administration of an oxytocin receptor antagonist into the lateral septum, by region‐specific conditional deficiency of the oxytocin receptor in the lateral septum or by inactivation of oxytocin neurones projecting to the lateral septum, suggesting that oxytocin neurones projecting to the lateral septum are critical for suppression of social fear conditioning in lactating mice.^96^

#### 2.3.5 Basolateral amygdala

Various studies have shown that oxytocin administration reduces amygdala activity in response to threatening social stimuli and, as a result, reduces anxiety in humans,^97^ although inconsistent reports^98^ and sexual difference^99^ have also been reported.

Administration of oxytocin into the basolateral amygdala before context‐shock pairings has been shown in rats to impair acquisition of context‐conditioned fear freezing and administration before re‐exposure to the context has been shown to suppress expression of conditioned freezing and facilitate its extinction in rodents.^100^ On the other hand, post‐session infusion of oxytocin into the basolateral amygdala has been shown to have no significant effects on consolidation of extinction learning.^100^ These findings suggest that the oxytocin receptor in the basolateral amygdala inhibits acquisition, suppresses recall and facilitates extinction of freezing behaviour in response to contextual fear conditioning, although contradictory results showing that oxytocin infusion into the basolateral amygdala enhances acquisition of contextual fear conditioning have also been reported.^79^

Oxytocin in the basolateral amygdala has also been shown to be involved not only in fear conditioning, but also in general discrimination learning. Microinjection of an oxytocin receptor agonist facilitates discrimination between conditioned signals for shocks and signals for the absence of shocks, being consistent with the view that oxytocin generally facilitates information processing of salient signals.^101^

#### 2.3.6 Central amygdala

Activation of the oxytocin receptor in the central amygdala has been reported to reverse isolation stress‐induced anxiety‐related behaviour in mice.^102^ Administration of oxytocin into the central amygdala has also been shown to reduce anxiety‐related behaviour in female rats.^103^ Oxytocin has also been suggested to reduce conditioned fear‐induced freezing behaviour in rats. It has been shown in rats that local administration of an oxytocin agonist activates oxytocin receptor‐expressing GABAergic inhibitory neurones in the lateral part of the central amygdala and, as a result, inhibits neurones in the medial part of the central amygdala projecting to the ventrolateral part of the periaqueductal gray, leading to reduction of freezing behaviour in response to contextual fear stimuli without affecting the cardiovascular fear response.^104^ Furthermore, activation of oxytocin fibres projecting to the central amygdala has been shown to suppress the expression of freezing behaviour in response to contextual fear stimuli.^3^ All of these findings suggest that oxytocin in the central amygdala reduces the expression of conditioned fear‐induced freezing behaviour.

Oxytocin in the central amygdala is involved in not only the expression of fear responses, but also the acquisition of fear learning. Administration of oxytocin agonists into the central amygdala before context‐shock pairing of fear conditioning training has been reported to impair the expression of freezing behaviour during re‐exposure to the context, suggesting that activation of the oxytocin receptor in the central amygdala impairs acquisition of contextual conditioned fear in rats.^79^

On the other hand, contradictory findings have also been reported. Pharmacological studies in rats have shown that administration of oxytocin into the central amygdala before recall of conditioned fear (context re‐exposure) enhances freezing behaviour, whereas administration of an oxytocin receptor antagonist suppresses freezing behaviour in rats, suggesting that the oxytocin receptor in the central amygdala facilitates the expression of conditioned freezing behaviour.^100^ Infusion of oxytocin after a recall session (extinction session) has also been reported to enhance freezing behaviour during the next recall session, suggesting that oxytocin impairs extinction of conditioned freezing.^100^ Consistent with the facilitative role of oxytocin in fear conditioning, administration of an oxytocin agonist into the amygdala has been reported to enhance fear acquisition in juvenile rats.^105^

The discrepant findings regarding the role of central amygdala oxytocin in the control of fear responses remain to be clarified. However, the role of oxytocin may not be as simple as oxytocin directly facilitating or inhibiting fear responses. It has been proposed that oxytocin contributes to the selection of active coping behaviour rather than passive defensive behaviour toward an imminent threat. Oxytocin receptor‐expressing GABAergic neurones in the lateral part of the central amygdala innervating neurones of the medial division of the central amygdala play an important role in switching between active and passive responses to an imminent threat. Blockade of the oxytocin receptor in the central amygdala has been shown to reduce active escape behaviour and increase passive freezing behaviour, whereas activation of the oxytocin receptor has been shown to increase active escape performance and reduce freezing behaviour in rats.^106^

#### 2.3.7 Bed nucleus of the stria terminalis

The bed nucleus of the stria terminalis plays an important role in neuroendocrine, autonomic and behavioural responses to an ambiguous threat or anxiety^107^, ^108^, ^109^ or those to discrete fear stimuli.^110^ Administration of an oxytocin receptor antagonist into the bed nucleus of the stria terminalis has been reported to impair acquisition of cued fear conditioning in a fear‐potentiated startle paradigm in rats, suggesting a facilitative role of the oxytocin receptor in the bed nucleus of the stria terminalis in acquisition of fear conditioning.^111^ On the other hand, oxytocin has been shown to reduce background anxiety in a fear‐potentiated startle paradigm in rats.^112^ Thus, the oxytocin receptor in the rat dorsolateral bed nucleus of the stria terminalis has been proposed to facilitate responses toward predictable, signalled fear but reduce responses to unsignalled threats or background anxiety via facilitating accurate discrimination between signals for threat and safety.^113^

The oxytocin receptor has also been shown to be involved in social avoidance after social defeat in female mice. Local administration of an oxytocin receptor antagonist into the anteromedial bed nucleus of the stria terminalis has been reported to increase the time spent for social interaction in socially defeated female California mice,^12^ suggesting that the oxytocin receptor in the bed nucleus of the stria terminalis facilitates social avoidance in socially defeated female mice.

#### 2.3.8 Dorsal or median raphe serotoninergic nucleus

Serotoninergic neurones in the raphe nucleus express the oxytocin receptor. Activation of the oxytocin receptor in the median raphe nucleus has been shown to facilitate serotonin release, and oxytocin‐induced anxiolytic action is impaired by a serotonin 2A/2C antagonist, suggesting that oxytocin reduces anxiety‐related behaviour via facilitation of serotonin release,^114^ although it has been shown that oxytocin receptor deficiency in serotoninergic neurones has no significant effect on anxiety‐related behaviour but reduces aggressive behaviour in male mice.^115^

In summary, oxytocin in rodents has been shown to reduce anxiety‐related behaviours via acting on the medial prefrontal cortex, hypothalamic paraventricular nucleus, central amygdala and raphe nucleus; to attenuate depression‐like behaviour by acting on the nucleus accumbens; and to reduce neuroendocrine stress responses by acting on the hypothalamic paraventricular nucleus. Oxytocin has also been shown to reduce conditioned fear‐induced freezing behaviour by acting on the medial prefrontal cortex, basolateral amygdala and central amygdala. On the other hand, oxytocin has been shown to augment conditioned fear‐induced freezing behaviour, conditioned fear‐induced potentiation of startle responses or social avoidance by acting on the central amygdala or bed nucleus of the stria terminalis. It is likely that oxytocin suppresses or augments stress responses depending on the situation by acting on different brain regions.

### 2.4 Influence of early‐life experiences on stress responses

Oxytocin has been shown in laboratory animals to be involved in effects of childhood experiences on stress responses in adulthood. Expression of the oxytocin receptor is influenced by environments. An enriched environment has been shown to increase expression of the oxytocin receptor in the prefrontal cortex, anterior insula and basolateral amygdala in prairie voles.^116^ On the other hand, deprivation of early maternal care in rhesus macaques has been reported to suppress expression of the oxytocin receptor in the hippocampus via epigenetic regulation and induce higher separation anxiety, which was rescued by a certain oxytocin receptor gene single nucleotide polymorphism (SNP).^117^ Neonatal maternal separation has also been shown to induce larger responses to noxious stimuli in an oxytocin‐ and histone deacetylase‐dependent manner in rats.^118^ All of these reported findings suggest that expression of the oxytocin receptor is influenced by early experiences and modulates stress responsiveness in adulthood.

Oxytocin has been reported to play an important role in early experience‐dependent development of the sensory cortices in mice.^119^ Activation of the oxytocin receptor during a developmental period has also been shown to have epigenetic actions to modulate social behaviour and sensory functions.^120^ Prenatal activation of the oxytocin receptor has been shown to reduce the aggressiveness of male mice in adulthood.^121^ In female prairie voles, early adversity induced by repeated daily isolation over the first 2 weeks of life has been reported to induce deficits in partner preference in animals with lower oxytocin receptor density of the nucleus accumbens, and neonatal stimulation of oxytocin release has been shown to buffer against the effects of early social isolation.^122^ All of these findings suggest that activation of the oxytocin receptor in early life has plastic and organisation actions.

In humans, oxytocin‐oxytocin receptor systems have been suggested to be involved in developmental plasticity, especially as a result of early experiences. Childhood abuse has been reported to induce lower plasma oxytocin concentrations in males^123^ and lower CSF oxytocin in females.^124^ Carriers of the G‐allele of the oxytocin receptor gene SNP rs53576, a SNP in the third of four introns, have been reported to show higher emotional empathy,^125^ while carriers of the A allele show lower empathy and higher vulnerability to childhood maltreatment,^126^, ^127^, ^128^ possibly as a result of difficulties in taking appropriate stress‐coping behaviour. However, opposite results showing that G‐allele carriers have higher scores of depression and are more sensitive to childhood trauma have been obtained in other studies.^129^

## 3 OXYTOCIN AND FOOD INTAKE

### 3.1 Activation of oxytocin neurones by food intake

Food intake or gastric distension has been shown to activate oxytocin neurones in the hypothalamus and facilitate oxytocin release,^130^, ^131^, ^132^, ^133^ whereas fasting reduces oxytocin mRNA.^134^ Ingredients of food appear to be important for activation of oxytocin neurones. Oral injection of sucrose^135^ and intragastric ingestion of sweetened condensed milk, although not that of high‐fat cream,^136^ have been reported to activate oxytocin neurones in the hypothalamus. Although the detailed mechanisms for this macronutrient‐selective activation remain unclear, fibroblast growth factor 21 (FGF21) has been suggested to be involved. Sucrose ingestion has been shown to release FGF21 from the liver in rodents and humans, resulting in suppression of sucrose preference.^137^, ^138^, ^139^ β‐Klotho, which is the FGF21 co‐receptor, was expressed in hypothalamic oxytocin neurones, and peripheral FGF21 administration activated oxytocin neurones. Thus, FGF21 may contribute to activation of oxytocin neurones after sucrose ingestion.^140^

Activation of oxytocin neurones after food intake is mediated, at least in part, by noradrenergic projections to the hypothalamus from A2 noradrenergic neurones, especially a subpopulation of A2 neurones expressing PrRP in the nucleus tractus solitarius.^23^, ^131^ The PrRP/noradrenergic neurones are stimulated by activation of the cholecystokinin octapeptide (CCK)1 receptor on afferent neurones of the gastric vagus nerves. PrRP deficiency or destruction of noradrenergic inputs to the hypothalamus impairs the activation of oxytocin neurones. CCK1 receptor deficiency, PrRP deficiency and oxytocin receptor blockade have been shown to increase meal size, suggesting that the CCK‐PrRP‐oxytocin pathway plays an important role for termination of each meal.^131^ The roles of other pathways activated or factors released after food intake in activation of oxytocin neurones remain to be clarified.

In fact, not only CCK, but also other anorexigenic factors assumed to be released after food intake have been shown to activate oxytocin neurones. For example, oxytocin neurones of the supraoptic nucleus express glucokinase and the insulin receptor. Oxytocin neurones of rat hypothalamic explants have been reported to be activated in response to glucose and insulin.^141^ Oxytocin neurones have also been shown to be activated by leptin in rats,^142^ and reduction of leptin after fasting reduces hypothalamic oxytocin mRNA.^143^ Peripheral or central administration of glucagon‐like peptide 1 has been reported in rats to activate supraoptic nucleus neurones^144^ or hypothalamic paraventricular oxytocin neurones.^145^ α‐MSH has also been shown to facilitate dendritic oxytocin release but to decrease electrical activity of oxytocin neurones in rats.^26^, ^146^ Oestrogen treatment has also been reported to increase oxytocin mRNA and induce anorexia in rats.^147^ Among these anorexigenic substances, oxytocin has been suggested to be downstream of some anorexigenic substances to induce anorexia, including CCK,^148^, ^149^, ^150^ leptin,^151^, ^152^ α‐nesfatin‐1,^153^ α‐MSH^154^ and oestrogen.^147^

Consistent with the view that oxytocin neurones are activated after food intake, ghrelin, an orexigenic hormone that is released during fasting, has been reported to hyperpolarise the majority of oxytocin neurones in the hypothalamus,^155^ although i.c.v. administration of ghrelin has been reported to induce Fos protein expression in some oxytocin neurones.^156^ Relaxin‐3, an orexigenic peptide that has been suggested to induce stress‐induced hyperphagia,^157^ has also been shown to inhibit oxytocin neurones in the hypothalamus in rats.^158^

The sensitivity of oxytocin neurones in response to food intake may be different in light and dark periods. Percentages of oxytocin neurones activated by re‐feeding are similar during the night and daytime, although the amount of food intake during the night is larger than that during the daytime,^131^ being consistent with the view that oxytocin neurones are sensitive to food intake in the daytime, when food intake is low.

In summary, hypothalamic oxytocin neurones have been shown to be activated by food intake via activation of the vagal afferents‐PrRP/noradrenergic pathway and release of anorexic factors including gastrointestinal factors.

### 3.2 Roles of oxytocin in the control of food intake

Oxytocin has anorexigenic actions.^130^, ^146^, ^159^, ^160^ Oxytocin administration has been shown to reduce food intake in laboratory animals and humans.^161^, ^162^, ^163^ The anorexigenic effect of oxytocin has been shown to be stronger in diet‐induced obese rodents.^164^ However, a meta‐analysis revealed that the anorexigenic effect of oxytocin administration is not statistically significant in humans.^161^ SNPs of the gene encoding the oxytocin receptor have been shown to be associated with eating disorders including anorexia nervosa and bulimia nervosa.^165^

On the other hand, although oxytocin receptor‐deficient male mice show late‐onset mild obesity,^166^ it has been reported that oxytocin receptor deficiency,^166^ chemogenetic inhibition of hypothalamic paraventricular oxytocin neurones,^167^ genetically targeted ablation of oxytocin neurones^152^ and destruction of hypothalamic paraventricular oxytocin neurones^150^ do not significantly change the total amounts of food intake in mice. Photogenic or chemogenetic activation of hypothalamic paraventricular oxytocin neurones has also be shown to have no significant effect on food intake (re‐feeding) in mice,^168^, ^169^ although food intake induced by activation of agouti‐related peptide (AgRP) neurones is reduced by activation of oxytocin neurones.^168^ On the other hand, an oxytocin receptor antagonist^170^ or oxytocin receptor deficiency^131^ increases meal size, suggesting that oxytocin released by food intake plays an essential role for termination of each meal.

Endogenous oxytocin appears to suppress intake of a specific macronutrient, namely carbohydrate. Oxytocin has been shown to reduce preference for carbohydrates but not fat.^135^ Oxytocin deficiency,^171^ ablation of oxytocin neurones^150^ and an oxytocin receptor antagonist^172^ have been shown in mice to enhance intake of sucrose solutions but not intake of palatable liquid emulsions^173^ or a high‐fat diet, suggesting that oxytocin preferentially suppresses carbohydrate intake, although no significant preference has been reported in oxytocin receptor‐deficient mice.^174^

The discrepant findings regarding the actions of oxytocin in food intake remain to be explained. However, effects of oxytocin are dependent on social or metabolic contexts. Oxytocin has been shown to reduce sucrose intake in isolated male rats but not in rats that were allowed social contact with conspecifics.^135^ Oxytocin has been shown to increase food intake in certain conditions. Oxytocin increases rather than decreases food intake in pregnant rats.^175^ Oxytocin may increase food intake under stressful conditions by attenuating stress‐induced anorexia. In female prairie voles, social isolation‐induced reduction of sucrose intake has been shown to be recovered by oxytocin, although oxytocin has no significant effect on sucrose intake in control voles.^176^ Oxytocin has also been shown in mice to reduce novel environment‐induced suppression of food intake^177^ and to mediate repeated stress‐induced attenuation of delayed gastric emptying,^178^ thus possibly leading to recovery of food intake.

As we have seen, it is likely that endogenous oxytocin contributes to the termination of each meal. However, oxytocin neurones are not located in the main stream for food intake control. Two distinct neuronal populations in the arcuate nucleus, AgRP/neuropeptide Y (NPY) neurones, which induce food intake, and pro‐opiomelanocortin (POMC) neurones, which induce satiety, play pivotal roles in food intake. These two populations project to melanocortin‐4 receptor‐expressing neurones in the hypothalamic paraventricular nucleus. The melanocortin‐4 receptor‐expressing neurones regulating feeding have been shown in mice to be glutamatergic, and none^167^ or only a few (2%)^179^ of them express oxytocin, although magnocellular oxytocin neurones have been shown to express the melanocortin‐4 receptor in rats and appear to receive regulation by α‐MSH.^26^ The relationship between oxytocin neurones and the main target melanocortin‐4 receptor‐expressing neurones remains to be clarified.

Oxytocin not only terminates food intake, but also affects metabolisms of the body. Activation of hypothalamic paraventricular oxytocin neurones has been reported to increase oxygen consumption in mice.^169^ Destruction of oxytocin neurones and oxytocin receptor deficiency have been shown in mice to reduce energy consumption and reduce thermogenesis in cold environments.^150^, ^180^ These findings suggest that oxytocin facilitates energy consumption and it is likely that decreased energy consumption is a cause of late‐onset obesity in oxytocin receptor‐deficient mice.

Oxytocin has also been shown to act peripherally to control metabolic homeostasis.^146^, ^181^ Oxytocin has been reported to increase glucose uptake from muscle and adipose tissue, hepatic gluconeogenesis, insulin secretion from the pancreas, and lipolysis, leading to improved insulin sensitivity and lipid homeostasis. These metabolic actions of oxytocin indicate the possible therapeutic usage of oxytocin for treatment of obese or diabetic patients.

Food intake‐induced activation of oxytocin neurones may also have functions other than control of food intake and energy consumption. Oxytocin has been reported in rats and mice to contribute to post‐prandial sexual appetite,^182^ natriuresis^183^ and the inhibition of sodium intake^184^ and alcohol ingestion.^185^

In summary, oxytocin has been shown in mice and rats to suppress food intake, especially carbohydrate intake, terminate each meal and increase energy consumption. On the other hand, oxytocin has been reported in laboratory rodents to increase food intake in certain conditions including pregnancy and stressful conditions. Oxytocin neurones themselves are not the main direct target neurones of orexigenic AgRP/NPY neurones or anorexic POMC neurones and oxytocin does not appear to directly control activity of AgRP/NPY or POMC neurones (see the next section). The precise mechanisms by which oxytocin modifies food intake remain to be clarified.

### 3.3 Sites of action of oxytocin with respect to modulating food intake

Food intake is controlled largely by two anatomically and functionally overlapping systems: a homeostatic or metabolic process and a reward‐related process.^186^ The homeostatic process is controlled by signals concerning nutritional status or energy reserve status such as signals from the catabolic peptides leptin and insulin and from the anabolic hormone ghrelin. The reward‐related process is associated with hedonia (pleasure, “liking” the food) and incentive or motivational salience (reinforcement or “wanting” the food).^187^ Reward‐related intake is the consumption of palatable food in the absence of nutrient deficits. Food intake is also modulated by stress‐related emotional processes and by cognitive processes.^188^ Oxytocin‐oxytocin receptor systems have been suggested to be involved in all of these systems. The oxytocin receptor is located in the homeostatic system including the arcuate nucleus, hypothalamic paraventricular nucleus, dorsomedial hypothalamus, ventromedial hypothalamus, lateral hypothalamus, dorsal vagal complex and parabrachial nucleus, in the reward‐processing system including the ventral tegmental area, nucleus accumbens and ventral pallidum, in emotion processing systems including the amygdala, lateral septum, bed nucleus of the stria terminalis, ventromedial hypothalamus, periaqueductal gray and raphe nucleus, as well as in cognitive systems including the prefrontal cortex, anterior cingulate and insular cortex.^13^, ^114^

#### 3.3.1 Prefrontal cortex

Oxytocin has been suggested to reduce the reward value of food by facilitating cognitive processes of shifting attention from short‐term merits to long‐term health consequences. Oxytocin administration with instruction to consider long‐term effects of food intake has been shown in female humans to induce changes in activity of the prefrontal cortex and reduce food craving.^189^ Oxytocin has also been shown to enhance prefrontal cortex activity and reduce food intake in fasted males.^190^

#### 3.3.2 Nucleus accumbens and ventral tegmental area

Oxytocin neurones of the hypothalamic paraventricular nucleus have been shown to project to the ventral tegmental area and nucleus accumbens and to regulate activity of dopamine neurones and dopamine release, resulting in control of reward‐related behaviours.^191^, ^192^, ^193^, ^194^, ^195^

Oxytocin administration into the ventral tegmental area^192^ and in the nucleus accumbens core^196^ has been shown to decrease deprivation‐induced food intake and palatable sucrose intake in rats.^197^

Oxytocin has also been shown to inhibit addiction by acting on the nucleus accumbens.^198^ It has been shown that i.c.v. administration of oxytocin blocks methamphetamine‐induced turnover of dopamine in the striatum or nucleus accumbens of mice^199^ and that administration of oxytocin into the nucleus accumbens reduces methamphetamine‐induced conditioned place preference, drug‐seeking behaviour in rats^200^ and ethanol‐induced conditioned place preference in mice.^201^ All of these findings suggest that oxytocin impairs the reward value of addictive drugs and ethanol.

On the other hand, oxytocin has been shown in laboratory rodents to facilitate social reward by acting on the ventral tegmental area or the nucleus accumbens via facilitation of dopamine release.^193^, ^194^, ^202^, ^203^

Oxytocin has been shown to enhance tonic activity of dopamine neurones of the ventral tegmental area in mice.^191^, ^193^ The differential actions of oxytocin to control the activity of dopamine neurones in response to addictive drugs and palatable food and to control the activity of dopamine neurones in response to social behaviour remain to be clarified. However, the effects of oxytocin on activity of dopamine neurones might be different depending on subtypes of dopamine neurones. Oxytocin has been shown in mice to activate dopamine neurones in the ventral tegmental nucleus but to inhibit dopamine neurones in the substantia nigra pars compacta.^204^ Oxytocin has also been shown in mice to filter certain inputs to dopamine neurones in the ventral tegmental area by inhibiting excitatory synaptic transmission through retrograde endocannabinoid signalling. Thus, oxytocin selectively suppresses inputs that are controlled by the presynaptic cannabinoid receptor and, as a result, modulates the excitability of dopamine neurones in an input‐selective manner.^205^

Differential activation of dopamine receptors after intake of addictive substances and social behaviour may also be involved in the differential actions of oxytocin. Drug reward appears to be mediated by the dopamine D1 receptor and social reward appears to be mediated by the dopamine D2 receptor, although both pair bonding and drug addiction have been suggested to increase the dopamine D1 receptor in the nucleus accumbens, resulting in selective interest in bonded partners or in drugs.^206^ Partner preference induced by mating has been shown to be impaired by dopamine D2 receptor antagonists in the nucleus accumbens shell of female prairie voles^207^ and amphetamine‐induced conditioned place preference has been shown to be blocked by a dopamine D1 receptor antagonist in the nucleus accumbens of male prairie voles.^208^ Increased activation of the D2 receptor versus D1 receptor has been suggested to induce social rewards over drug rewards.^207^, ^208^

In humans, intranasal oxytocin administration has been shown to decrease activation of the ventral tegmental area in response to foods and to increase activities of the dorsal anterior cingulate cortex, frontopolar cortex,^165^ ventromedial prefrontal cortex, supplementary motor area, anterior cingulate, and ventrolateral prefrontal cortices,^190^ which are involved in cognitive functions.

In summary, oxytocin reduces the reward value of palatable food intake possibly by inhibiting dopamine release in the nucleus accumbens.

#### 3.3.3 Hypothalamus

The oxytocin receptor is located in various hypothalamic nuclei, including the dorsomedial hypothalamus, arcuate nucleus, ventromedial hypothalamus and lateral hypothalamus,^114^ which have been shown to be involved in the control of food intake and energy expenditure.^209^ Administration of oxytocin into the ventromedial hypothalamus has been reported to reduce food intake in rats.^210^ Chemogenetic activation of oxytocin receptor‐expressing glutamatergic neurones in the arcuate nucleus that project to the hypothalamic paraventricular nucleus has also been reported in mice to rapidly induce satiety, indicating the importance of oxytocin receptor‐expressing arcuate neurones in the control of food intake.^211^

#### 3.3.4 Amygdala

Oxytocin administration in the basolateral and central amygdala has been shown to suppress food intake moderately in rats.^212^ On the other hand, an oxytocin receptor antagonist has been reported in mice to impair acquisition of conditioned taste aversion, suggesting that oxytocin facilitates acquisition of conditioned taste aversion.^213^

#### 3.3.5 Dorsal vagal complex

Oxytocin neurones in the hypothalamic paraventricular nucleus project to the dorsal vagal complex that consists of the nucleus tractus solitarius and dorsal motor nucleus of the vagus (DMV). Oxytocin has been shown in rodents to act on DMV neurones to inhibit gastric motility, resulting in termination of a meal.^135^ On the other hand, under stressful conditions, it has been suggested that oxytocin has opposite functions in rats and increases gastric tone via suppression of GABAergic inhibitory inputs to DMV neurones, which has facilitative actions on gastric motility.^214^

In summary, oxytocin has been shown to act on homeostatic systems including the arcuate nucleus and dorsal vagal complex to terminate food intake, on reward systems including the ventral tegmental area and nucleus accumbens to reduce food reward, and on cognition systems including the prefrontal cortex to facilitate cognitive control of food intake.

### 3.4 Interactions between stress and food intake

Stress responses are influenced by bodily metabolic status.^215^, ^216^ Metabolic information is transmitted via neurones or hormones to the hypothalamus, which controls bodily metabolisms and stress responses. Fasting reduces anxiety‐ or fear‐related behaviours. Reduction of anxiety or fear contributes to induction of proactive and risky food‐seeking foraging behaviours during a negative energy balance. On the other hand, perceived stress and glucocorticoid release have been shown to be reduced after acute intake of high carbohydrate or palatable food in humans.^188^, ^217^ Stress‐vulnerable individuals may thus learn to self‐medicate comfort food,^218^ resulting in increased intake of palatable food under stressful conditions.

Stressful stimuli have been shown to modulate food intake and energy expenditure.^215^, ^219^ Under stressful conditions such as fight or flight, food intake is suppressed and energy expenditure is increased as a result of elevated sympathetic tones, leading to a negative energy balance. However, in some stressful conditions, hyperphagia^220^, ^221^ and/or hypothermia,^222^ which result in a positive energy balance, are induced. In humans, an increase in perceived stress has been shown to be associated with an increase in high‐fat/carbohydrate palatable food intake^188^, ^218^, ^223^ and visceral obesity.^224^

The detailed mechanisms of interactions between stress and energy metabolisms remain unclear. However, the involvement of AgRP/NPY neurones has been suggested. Fasting activates AgRP/NPY neurones in the arcuate nucleus, which not only induce food intake, but also are suggested to reduce fear‐related behaviour.^225^, ^226^, ^227^ Arcuate AgRP neurones project to the medial amygdala neurones that innervate the posterior bed nucleus of the stria terminalis, and this AgRP‐medial amygdala‐bed nucleus of the stria terminalis pathway has been shown to be involved in fasting‐induced behavioural changes in mice.^226^ AgRP/NPY neurones also project to the periaqueductal gray, which is involved in fear and anxiety‐related behaviour. Furthermore, NPY has anxiolytic actions.^228^ Not only AgRP/NPY, but also the peripheral anorexic hormone leptin and the orexigenic hormone ghrelin have been suggested to be involved in modulation of neuroendocrine stress responses during fasting or satiated conditions. Leptin has been shown to inhibit noradrenaline release in the hypothalamic paraventricular nucleus and adrenocorticotrophic hormone release in response to stressful stimuli, whereas ghrelin facilitated these stress responses in rats.^229^ On the other hand, the stress‐related hormones CRH and glucocorticoids, and ghrelin have been suggested to be involved in the effects of stress on food intake. A CRH1 receptor antagonist has been reported to suppress stress‐induced eating in humans,^230^ and CRH neurones have been shown to induce preference for a high‐carbohydrate diet in mice.^231^ Glucocorticoid administration has also been shown to increase food intake, especially intake of carbohydrates and proteins, in humans^232^ possibly via activation of brain reward systems.^187^ Social defeat stress has been shown to facilitate ghrelin release and stress‐induced hyperphagia is impaired in ghrelin receptor‐deficient mice.^233^

Oxytocin has also been suggested to play a role in interactions between stress and metabolic homeostasis.

As we have seen, stressful stimuli and food intake activate oxytocin‐synthesising neurones, which modulate stress responses and food intake^160^ by acting on common brain systems including homeostasis, reward, emotion and cognition systems. It is possible that oxytocin contributes to stress‐induced food intake changes and food intake‐induced attenuation of stress responses. Patients with eating disorders frequently show emotional and social dysfunctions, which induce stressful situations and exacerbate symptoms of eating disorders. Genetic predispositions of oxytocin systems have been suggested to induce vulnerability to stressful stimuli and liability to overeating or eating disorders in humans.^165^, ^234^, ^235^, ^236^ A negative relationship between socioeconomic status and childhood obesity has been shown in A allele carriers of the oxytocin receptor gene SNP rs53576.^237^ A allele carriers with a low socioeconomic status have been shown to have a high body mass index. On the other hand, no significant association between socioeconomic status and body mass index was found in GG genotyped children. The differential susceptibility of childhood obesity to socioeconomic status is consistent with the view that the oxytocin receptor is involved in effects of adverse environments on energy metabolisms. The sites of action of oxytocin with respect to controlling aberrant eating during stressful stimuli remain to be determined. Oxytocin has been shown to modulate the activity of cognition‐related brain regions in humans.^98^ Oxytocin may act on the prefrontal cortex to modulate executive functions, to reduce cognitive rigidity and to suppress impulsivity, as been associated with the intake of palatable foods and obesity in humans.^238^ Oxytocin administration has also been shown to reduce eating‐related concern or attentional bias,^239^ to attenuate cognitive rigidity, and to decrease salivary cortisol^240^ in anorexia nervosa, suggesting that oxytocin reduces stress responsiveness related to food intake via improvement of cognitive functions.

## 4 ROLES OF OXYTOCIN IN THE CONTROL OF ADAPTIVE BEHAVIOURS

Oxytocin has been shown to control not only stress responses and food intake, but also social behaviours. Several hypotheses have been proposed to explain the actions of oxytocin in an integrative way.^241^ Studies on the role of oxytocin in reproduction‐related behaviours, including pair bonding,^206^, ^242^ mating and maternal behaviour of laboratory animals,^243^ as well as in human behaviours requiring higher cognitive functions, including trust and altruism, have led to the prosocial hypothesis that oxytocin facilitates prosocial behaviours in general.^244^ Oxytocin paired with charitable social cues has been shown in humans to reduce out‐group rejection even in xenophobic individuals, although the effects of oxytocin appear to be dependent on the experimental conditions.^245^

On the other hand, oxytocin has also been shown in humans to evoke antisocial behaviours in competitive situations^246^ and to induce defensive aggression toward out‐group members.^247^ Intranasal oxytocin application has also been shown to facilitate startle response to threatening stimuli in humans, consistent with the view that oxytocin increases the salience of threatening stimuli.^248^ In rodents, oxytocin mediates maternal aggression.^249^ Oxytocin has been shown to facilitate perceptual processing of olfactory signals^250^ by increasing the signal‐to‐noise ratio via the excitatory drive of inhibitory neurones and that of auditory signals^251^ and, as a result, enhance social behaviours in rodents. Oxytocin has been reported in rodents to be indispensable for induction of odour‐driven social avoidance from parasitised individuals^252^ or odour‐dependent learning, although not that of non‐social learning,^253^ which is either appetitive or aversive, via acting on the medial amygdala, piriform cortex, and anterior olfactory cortex, where olfactory sensory information is processed. Thus, the social salience hypothesis that oxytocin increases the salience of either positive or negative social stimuli by possibly facilitating sensory processing has been proposed.^69^, ^246^, ^254^

Consistent with the idea that oxytocin facilitates sensory processing of socially relevant stimuli, the oxytocin receptor is located densely in the olfactory system in rodents,^255^ whereas the oxytocin receptor is abundantly located in the visual system or visual attention‐related systems including the superior colliculus and nucleus basalis of Meynert in primates.^256^ Oxytocin may modulate most relevant sensory systems to regulate social behaviour dependent on species.^257^ Oxytocin has been shown to enhance eye gaze between humans^69^ and between dogs and their owners.^258^ Oxytocin administration in humans has also been shown to enhance recognition of emotion expressed in faces that are either fearful or happy.^254^

The oxytocin receptor is also located in somatic sensory systems including the trigeminal ganglion, dorsal root ganglion and dorsal horn of the spinal cord. Oxytocin is released after pleasant tactile stimuli in rats^259^ and it has been proposed to contribute to the formation of social bonding between mothers and their offspring via pleasant tactile stimuli.^260^ Subjective feelings induced by tactile stimuli are dependent on the social relationship between receivers and givers of touch stimuli. Oxytocin released by intimate social interactions may modulate sensory processing of tactile stimuli to induce a comfort sensation.

The social salience hypothesis, however, cannot fully explain the finding that oxytocin appears to preferentially increase the salience of positive social cues rather than negative social cues.^261^ Oxytocin has been shown to enhance activity of the amygdala in response to happy facial expressions but to attenuate the activity toward fear faces^262^ and to improve recognition of happy facial expression selectively.^263^

Activation of the social reward system may be involved in preferential facilitation of positive social information processing. Oxytocin facilitates dopamine release in response to socially relevant cues in order to evoke positive social behaviours.^193^, ^204^, ^264^, ^265^ Interactions with serotoninergic projections have been shown to play an important role. Within the nucleus accumbens, the oxytocin receptor is located on presynaptic axon terminals of dorsal raphe serotoninergic neurones, and serotonin released by oxytocin has been shown in mice to promote long‐term depression of glutamatergic transmission via the 5HT1b receptor in the nucleus accumbens to induce social reward.^266^

Thus, oxytocin increases the reward value of social behaviour (social reward hypothesis) and, as a result, preferentially enhances salience of positive emotional stimuli. The facilitative action of oxytocin on dopamine release appears to be selective to social situations. Oxytocin reduces dopamine release in response to addictive drugs and possibly in response to food, and oxytocin administration has been examined as a therapeutic tool for addiction or obesity.^198^ The detailed mechanisms for this selective action of oxytocin remain to be clarified.

Oxytocin has also been suggested to facilitate approach behaviour to positive stimuli and to suppress withdrawal behaviour to negative stimuli, regardless of whether they are social or non‐social stimuli (the approach/withdrawal hypothesis).^267^ Oxytocin has been proposed to act on the prefrontal cortex‐amygdala circuit controlling fear or threat responses to attenuate behavioural avoidance or autonomic stress responses in response to negatively valenced stimuli, whereas, at the same time, oxytocin acts on the dopaminergic reward system to increase approach behaviour in response to emotionally salient stimuli including not only stimuli inducing positive emotion, but also stimuli inducing negative emotion such as anger, envy, and gloating,^268^ although the role of the dopaminergic system in the control of approach responses to negative emotion‐related stimuli remains to be established.

On the other hand, oxytocin has been suggested to facilitate active coping behaviour in certain conditions and to switch from passive stress‐coping behaviour to active stress‐coping behaviour.^24^ Recently, oxytocin has been shown to reduce passive coping behaviour such as freezing behaviour by acting on neurones in the lateral part of the central amygdala, which inhibits the activity of periaqueductal gray‐projecting central amygdala neurones, and, as a result, induces active escape behaviour^106^ or active defensive behaviour.^269^ Consistent with this idea, oxytocin has been shown to suppress social loss‐induced passive coping behaviour in male prairie voles^84^ and to induce social defeat‐induced risk assessment behaviour (head orientation) in female mice by acting on the bed nucleus of the stria terminalis.^12^, ^270^ Furthermore, oxytocin facilitates expression of social defeat posture, which is an active coping behaviour showing subordination toward dominant conspecifics, by possibly acting on the ventromedial hypothalamus or periaqueductal gray in male mice.^13^ In rats, the oxytocin receptor in the insular cortex has been shown to be indispensable for showing approach behaviour to distressed juvenile conspecifics and for showing avoidance behaviour to distressed adult conspecifics, suggesting the importance of the oxytocin receptor in appropriate active coping behaviours.^271^ All of these findings suggest that oxytocin facilitates active stress‐coping behaviour by acting on multiple regions.

These hypotheses are not mutually exclusive. The oxytocin receptor is located in various brain sites, and oxytocin has multiple actions depending on the situation or the individual by acting on various brain sites for an appropriate decision to be made depending on the social environment.^272^

## 5 CONCLUSIONS

**Figure 1:**
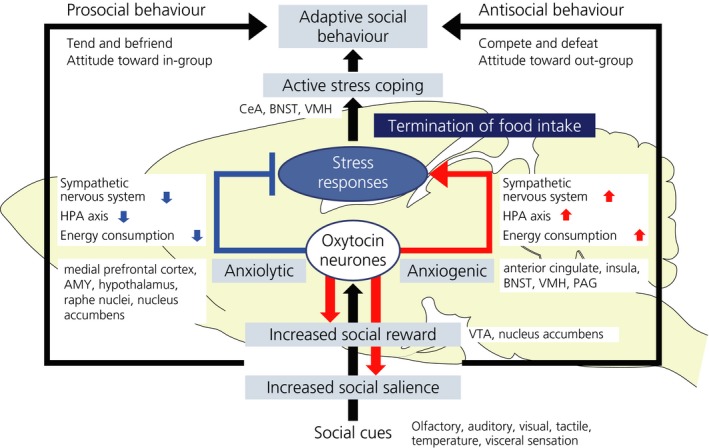
Oxytocin facilitates sensory processing of socially salient stimuli, facilitates reward value of social behaviours, modulates stress responses depending on the situation, induces active coping behaviour and terminates food intake. Oxytocin acts on various brain regions to play multifaceted roles leading to adaptive behaviours. AMY, amygdala; BNST, bed nucleus of the stria terminalis; CeA, central amygdala; HPA, hypothalamic‐pituitary‐adrenal; PAG, periaqueductal gray; VMH, ventromedial hypothalamus; VTA, ventral tegmental area

It is essential for group‐living mammals to find appropriate partners, to bond with and mate with partners, to create and nurture their offspring, to build up friendship, to make an in‐group society, and to deal with difficulties cooperatively under stressful conditions. Oxytocin appears to be involved in these steps to some degree by acting on various brain regions. Oxytocin facilitates sensory processing to preferentially receive socially salient signals and facilitates reward values of prosocial behaviour, especially toward in‐group members and, at the same time, reduces anxiety and induces satiety, resulting in appropriate active coping behaviours being taken to adapt to social environments (Figure 1). Reproduction, food intake, stress responses and social behaviours are associated with each other, and it is interesting to speculate that oxytocin modulates these functions in an integrative way to induce active and adaptive coping behaviours.

In stressful conditions, there are at least two aspects of behavioural strategies. One is the “tend‐and‐befriend” or “fight or fright” aspect^273^ and the other is passive coping behaviour (freezing, withdrawal) or active coping behaviour (active escape, flight, or fight). Oxytocin appears to facilitate the tend‐and‐befriend strategy in non‐competitive situations, whereas it facilitates fight or flight behaviours in competitive situations. Oxytocin also appears to facilitate active coping behaviour rather than passive behaviour. Because behavioural or neuroendocrine stress responses are final common outputs after integrating various processes of internal and external information, oxytocin may induce augmentative or attenuated stress responses depending on experimental situations by acting on multiple brain areas.

However, the detailed mechanisms of the actions of oxytocin remain unclear. It is not known how oxytocin induces apparently opposite behaviours depending on the situation. The detailed functional and anatomical heterogeneity of oxytocin neurones remains to be elucidated. The mechanisms of gender difference^274^, ^275^ should also be clarified. Oxytocin has also been reported to act not only on the oxytocin receptor, but also on vasopressin receptors, on δ subunit‐containing GABA_A_ receptors^276^ and on transient receptor potential vanilloid (TRPV1).^277^ Although pharmacological studies have shown the importance of vasopressin receptors in the actions of oxytocin,^278^ molecular and genetic evidence directly showing the roles of vasopressin receptors is not sufficient.^279^, ^280^ The physiological roles of these receptors in the actions of oxytocin remain to be clarified. Further studies are also necessary to facilitate therapeutic potentials of oxytocin^281^, ^282^ or oxytocin‐related treatments including diet ingredients^283^ and intestinal microbial symbionts.^284^
